# Dynamic Adsorption of H_2_S onto a Goethite-Based Material

**DOI:** 10.3390/molecules27227983

**Published:** 2022-11-17

**Authors:** Francisco Jose Alguacil, Manuel Ángel Alonso, Félix Antonio López, Jose Ignacio Robla

**Affiliations:** Centro Nacional de Investigaciones Metalúrgicas (CENIM-CSIC), Avda. Gregorio del Amo 8, 28040 Madrid, Spain

**Keywords:** hydrogen sulfide, adsorption, desulfurization, iron oxide, column tests

## Abstract

The use of adsorption technology to remove H_2_S from synthetic gas (H_2_S and N_2_) using a goethite-based adsorbent was investigated. The influence of the H_2_S feed concentration (150–600 mg), the adsorbent dosage (1–4 g), and the gas flow rate (210–540 cm^3^/min) on the breakthrough curves and H_2_S adsorption on the adsorbent at the breakthrough point was investigated. Dynamic column experiments were performed to provide data for the theoretical models and to verify the performance of the system in the adsorption process. The theoretical models used in the present work were found to predict the adsorption breakthrough performance reasonably well.

## 1. Introduction

Biogas is mostly produced by anaerobic digestion or different types of wastes and its use for domestic purposes, such as heating, e.g., cooking and lighting, was widely known in Asian and African countries [1]. Thanks to its high methane content (generally 50% to 70%), biogas can substitute natural gas in many applications such as heat and electricity generation for households and industries, biofuel for vehicles, and injection into the gas grid, so production of biogas has augmented in Europe and other countries and is now identified as priority objective by the European Industrial Biomass Initiative (EIBI), the European Energy Research Alliance (EERA), and the Strategic Energy Plan (SET) plan.

Concerns about climate change and the depletion of the available oil and gas reserves prompted a broad discussion on the use of biogas as a clean renewable energy resource, so biogas is more and more playing a key role in many applications being a renewable substitute for natural gas. Countries such as Germany, Sweden, and the UK are pushing strongly with solid support from the industry and national subsidies. Spain is still in its beginnings, with only one industrial biogas production plant located in Valdemingómez, Madrid, but interest and expectations are increasing, based on the initiatives of the Spanish Ministry for Ecologic Transition and Demographic Challenge (MITECO) for publishing a Biogas Roadmap for 2030 and also the interest of waste treatment companies, which see biogas as a profitable outcome from the wastes in addition to reducing waste disposal costs. Moreover, according to a recent study performed by Ecofys [2], it is possible to scale up renewable gas production between now and 2050 to more than 120 billion cubic meters annually, including both renewable hydrogen and biomethane. This increasing availability of biogas could facilitate multiple applications, such as its use in existing gas infrastructure for the heating of buildings, to produce dispatchable electricity as a complement to wind and solar, and to fuel heavy transport, which could save in all about EUR 140 billion annually by 2050 compared to a future energy system without any gas [3].

In addition to CH_4_ and CO_2_, which are the main components of biogas, a large variety of adverse compounds in trace form (sulfur-containing compounds, silicon, halogens, NH_3_, siloxanes, mercaptans, etc.), are hampering its wider application [4]. One of the main contaminants, sulfur, is often present in biogas in the form of H_2_S in amounts ranging from a hundred ppm to more than 5%, which makes it necessary to remove it before use [5].

The use of biogas cleaning technologies are intrinsic to the use of biogas, even in more permissive combustion engines and the most widely used methods include adsorption, absorption, and membrane-based gas separation processes. Among these purification methods, the adsorption process tends to be the most efficient due to its simplicity of design, ease of operation, and insensitivity to toxic substances. However, when defining the cleaning strategy, two main issues have to be taken into account: biogas composition (especially contaminants) and the final use of the biogas. The selection of cleaning strategies will be also determined by the thermal balance of the process, and this is especially important in some applications of biogas.

Different types of sorbents (raw materials and wastes) have been studied as sorbents for biogas cleaning [6,7,8,9,10,11]. One of the most investigated materials is biochar and the targeted contaminant is H_2_S. Recent studies [12] reported that H_2_S adsorption capacities of biochar (0.18–1.38 mg/g) are much lower than commercial sorbents, while the performance of ashes is still too poor (0.05–0.38 mg/g). Zeolites (5A and 13X) are also other intensively considered candidates for biogas cleaning and for those cases, hydrophobic zeolites are considered more adequate. In a recent study, zeolite recovered from tuff waste obtained removal efficiencies of up to 40 mg/kg [13]. Investigation of specific properties and nature of the materials appear as a significant challenge to optimize their performance for the tailored contaminants. They have also demonstrated their efficiency in desulfurisation at moderate temperatures (300–500 °C) of Zn–Mn spinels obtained by the synthesis of black mass from mechanical recycling of alkaline batteries [14].

Hydrogen sulfide reacts with iron hydroxide or oxide to form iron sulfide (FeS). When the material is saturated, it can either be regenerated or changed. Sorbents based on Fe or Fe-based oxides are relatively cheap; some types are environmentally friendly and their capacity for simultaneous adsorption of more than one impurity has also been reported in the literature. For example, the use of iron oxide-based sorbents for simultaneous capture of H_2_S and Hg was demonstrated for simulated syngas at temperature ranges of 60–140 °C. For these types of materials, H_2_S removal efficiencies as high as 99% and an adsorption capacity of 21%wt has been reported [15], but the chemisorption mechanism observed for H_2_S removal could explain the low efficiency of the regeneration process.

With the aid of activated carbon, a series of mesoporous ZnO/SiO_2_ adsorbents are fabricated by the sol–gel method [16]. The usefulness of this adsorbent is attributed to activated carbon which has two roles: anchor and confine ZnO to obtain highly dispersed small nanoparticles and to produce more oxygen vacancies in ZnO to facilitate lattice diffusion.

An adsorbent material consisting of a specific composition of Cu, Mg, and Al oxides is used in the removal of this toxin H_2_S [17] and, whereas copper oxide (CuO) is the active adsorbent and is transformed into CuS, aluminum and magnesium oxides contribute to adsorbent activity and stability.

A tuned composite structure of Cu, Zn, Fe, and Al elements is also used in adsorbent investigations to reduce H_2_S emissions [18]. The combination of the above elements in a composite crystallinity structure resembling hydrotalcite or aurichalcite type-materials showed excellent H_2_S uptake.

Iron(III) oxide and titanium(IV) oxide have been used in the removal of hydrogen sulfide from biogas [19]. In the investigation, both oxides are used separately or in different combinations. The effectiveness in the removal of H_2_S by these oxides is demonstrated.

CeO_2_/Fe_2_O_3_,CeO_2_/Mn_2_O_3_, and CeO_2_/Mn_2_O_3_/Fe_2_O_3_ nanocomposites are other examples of adsorbents for the removal of H_2_S from a gas flow [20]. From the above composites, the adsorbent containing the mixture of cerium, manganese, and iron oxides presented the maximum adsorption capacity.

In this paper, the adsorption under dynamic conditions of mimic H_2_S (provided from a mixture of H_2_S and N_2_) by a micronized goethite mineral is investigated. Different experimental variables are studied: adsorbent dosage, gas mixture flow, and H_2_S concentration being the experimental data fit to various models. Moreover, the adsorption efficiencies of the fixed bed, under the above experimental conditions, are also given. 

## 2. Results and Discussion

**Figure 1:**
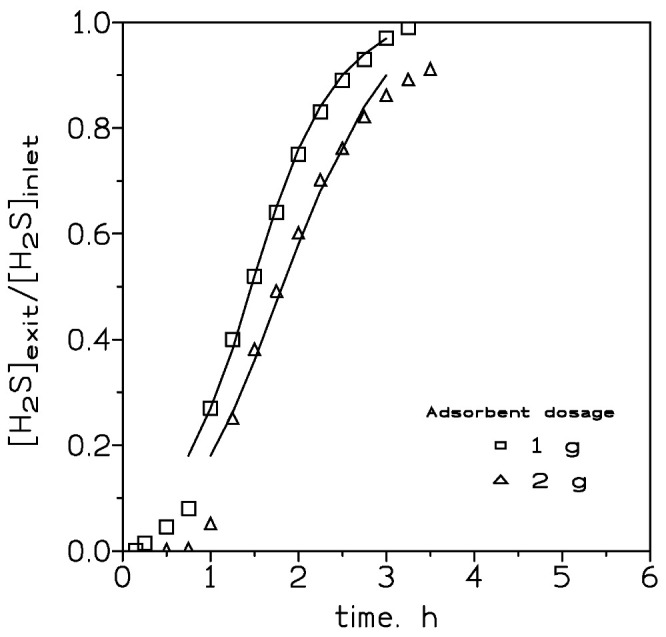
Experimental (points) and model (lines) breakthrough curves at various adsorbent dosages. H_2_S dosage: 400 mg. Gas flow: 210 cm^3^/min. Temperature: 20 °C.

Figure 1 showed the variation in the [H_2_S]_exit_/[H_2_S]_inlet_ relationship versus time using different adsorbent dosages. It can be seen that the increase in the adsorbent packed into the column had an influence on the breakthrough curves, both in terms of shifting the curve to the right and also in the shape of the corresponding curve.

In Table 1, the estimated H_2_S loading values, at the breakthrough point, were given, and under the present experimental conditions no variation in the H_2_S loading onto the adsorbent was found at the various adsorbent dosages. In the first instance, the adsorption of H_2_S on this adsorbent can could be related to the moving boundary or shrinking core model, thus, the mass transfer across the adsorbent shell particle dominated and the H_2_S adsorption rate decreased as the thick of the shell increased. If the driving force was especially small, the resistance encountered by the shell to the mass transfer increased to the point that the yield of a full breakthrough curve was not possible, being this probably the situation encountered when an adsorbent dosage of 4 g was used.

**Figure 2:**
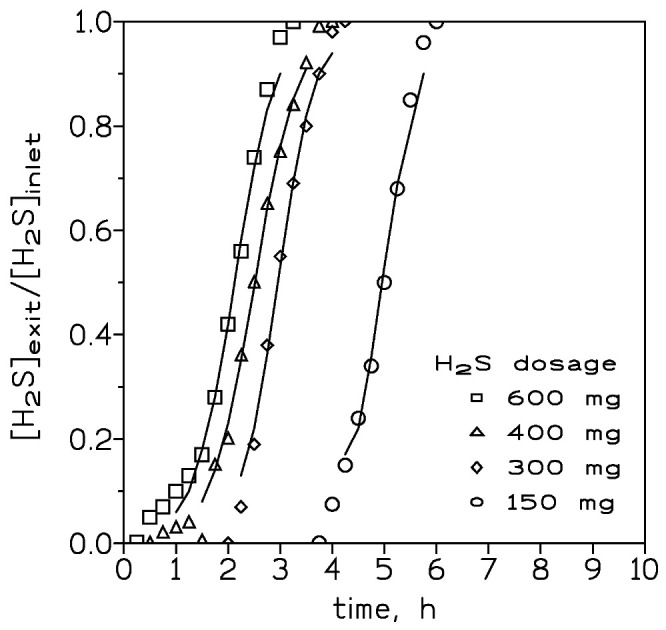
Experimental (points) and model (lines) breakthrough curves at various inlet H_2_S dosages. Adsorbent dosage: 4 g. Gas flow: 420 cm^3^/min. Temperature: 20 °C.

Figure 2 presented the breakthrough curves measured at various inlet H_2_S dosages ranging from 150 to 600 mg, a temperature of 20 °C, and a gas flow of 420 cm^3^/min. It was shown that the slope of the curves became fairly similar, and the period time between reaching the breakthrough point and the time at which the column was fully loaded increased due to an increase in the inlet H_2_S dosage. Thus, the mass transfer zone in the column became longer at greater inlet H_2_S dosages. At the breakthrough point, the H_2_S loading onto the adsorbent at the various adsorbate inlet concentrations was given in Table 2.

It was shown that there was not a direct relationship between these two variables: (i) inlet H_2_S dosage and (ii) H_2_S concentration at the breakthrough point. This lack of relationship between points (i) and (ii) can be attributable to two causes: the very similar slopes resulting from the four inlet H_2_S dosages and the increase in the time elapsed between the breakthrough point and the bed saturation point, with an increase in the inlet H_2_S dosage to the column.

**Figure 3:**
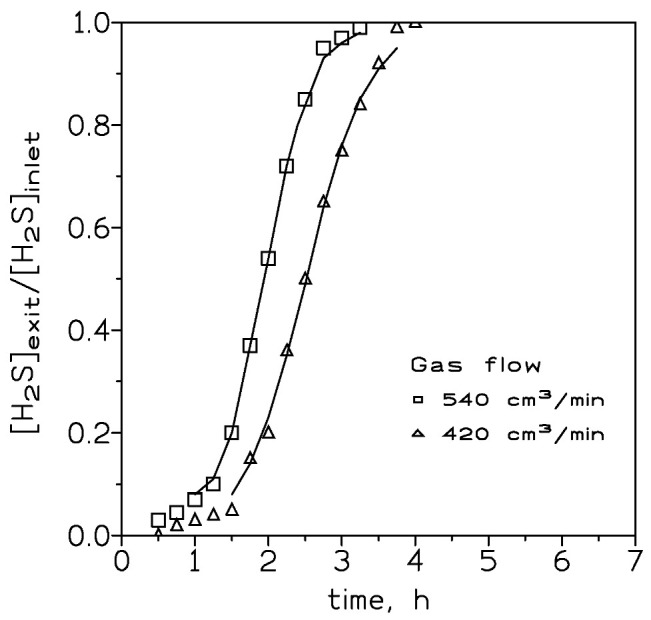
Experimental (points) and model (lines) breakthrough curves at various gas flows. H_2_S dosage: 400 mg. Adsorbent dosage: 4 g. Temperature: 20 °C.

At an inlet H_2_S dosage of 400 mg and adsorbate dosage of 4 g (bed volume of 3.8 cm^3^), the effect of the gas stream flow rate on the H_2_S loading onto the adsorbent was investigated. Figure 3 showed the results. It was clear that it took a longer time for the adsorbent bed to reach the breakthrough point at a lower gas flow. Moreover, the shape of the loading curve became less steep with the decrease of the gas flow. Table 3 shows the data about H_2_S uptake onto the adsorbent at these three flows.

In theory, steeper breakthrough curves may be an indication of greater H_2_S loading onto the adsorbent; however, these results are against what is said above. As it was mentioned in the literature [21], the breakthrough curves reflected what occurred in the mass transfer zone of the column. If the adsorption process was of an irreversible nature, as it was being the present study about the reaction of H_2_S onto a Fe_2_O_3_-based adsorbent, before reaching the breakthrough point there was always a zone of pristine adsorbent which resulted in a mass transfer zone always reacting with the inlet gas flow. Thus, the gas stream exiting the column did not contain any H_2_S; the reaction rate was represented by the overall rate corresponding to the mass transfer zone. As the column was being loaded with H_2_S, the mass transfer zone shifted to the column bottom; the adsorbent was fully loaded with H_2_S, and H_2_S began to exit the bed. Considering that the overall reaction rate was infinite, a thin layer of adsorbent was used to be the mass transfer zone, and the exiting H_2_S dosage reached the inlet value almost instantly. In the above conditions, the breakthrough curve adopted a near vertical form in the [H_2_S]exit/[H_2_S]inlet versus time profile.

The above behavior was less frequent, since the reaction rate occurring in the mass transfer zone tended to be finite As a consequence, the shape of the curve becomes less steep as the overall reaction rate in the mass transfer zone becomes slower. The results shown in Figure 3 indicated that a greater flow rate was accompanied by a greater reaction rate; this may be indicative that the process was in a mass transfer control regime.

As it was mentioned above, an increase in the thickness of the particle shell resulted in a decrease in the adsorbate adsorption rate, and under this circumstance, the breakthrough curve was steeper at shorter times, decreasing the slope when the elapsed time increased.

**Figure 4:**
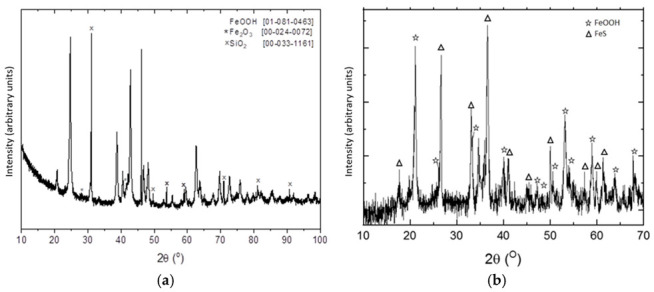
XDR diagrams of the pristine (**a**) and after H_2_S uptake (**b**) adsorbent.

Figure 4 showed the XRD pattern of the material before and after H_2_S adsorption. The presence of FeS can be observed in the resulting material after H_2_S uptake onto the adsorbent, which was evidence of the chemical reaction between goethite and H_2_S during the adsorption process. However, the presence of goethite in this final material was also evidence that the complete saturation process had not yet ended; this situation can be attributable to the diffusional resistances encountered during the adsorption process halted the complete reaction between goethite and H_2_S, because they hindered the further access of H_2_S to the interior of the adsorbent particle.

In a similar manner to other unit operations, adsorption is a process where success is affected by a series of operational parameters, i.e., adsorption capacity and mass transfer rate. In dynamic form, and at first instance, prediction of column performance can be performed using a macroscopic version to describe the behavior of an adsorbent packed in a column [22]. The equation describing the macroscopic model can be written as:

(1) $$\ln\left(\frac{{\left[S\right]}_{t}}{{\left[S\right]}_{\mathrm{inlet}}-{\left[S\right]}_{t}}\right)=K{\left[S\right]}_{\mathrm{inlet}}\left(t-\frac{{\left[S\right]}_{\mathrm{eq}}m}{{\left[S\right]}_{\mathrm{inlet}}f}\right)$$

where [S]_t_ is the solute concentration in the exit solution at time t, [S]_inlet_ is the inlet solute concentration, K is the rate constant of the process, [S]_eq_ the equilibrium solid-phase concentration of the adsorbed solute, m the mass of adsorbent, f the flow rate, and t the elapsed time. Accordingly, in the above expression the left-hand term is a linear function of time, being the whole equation the same as the simplified logistic function that expressed the biological growth and distribution, thus:

(2) $$\ln\left(\frac{{\left[S\right]}_{t}}{{\left[S\right]}_{\mathrm{inlet}}-{\left[S\right]}_{t}}\right)=K\left(t-t_{50}\right)$$

The two parameters K (rate constant) and t_50_ (elapsed time at which 50% of the solute is adsorbed onto the adsorbent) in the above equation can be estimated from the slope and intercept, respectively, of a plot of the left-hand term in Equation (2) versus time. Such parameters for the various experimental conditions used in the present investigation are listed in Table 4. Using these estimated values, the breakthrough curves for these experimental conditions may be predicted for H_2_S adsorption on the iron oxide-based adsorbent. Figure 1, Figure 2 and Figure 3 compared the predicted and experimental data for different adsorbent doses, H_2_S doses, and flow rates, respectively. It can be observed that the model compared reasonably well with the experimental data. Thus, Equation (2) can be used for the representation of the column adsorption process under the present experimental conditions.

**Figure 5:**
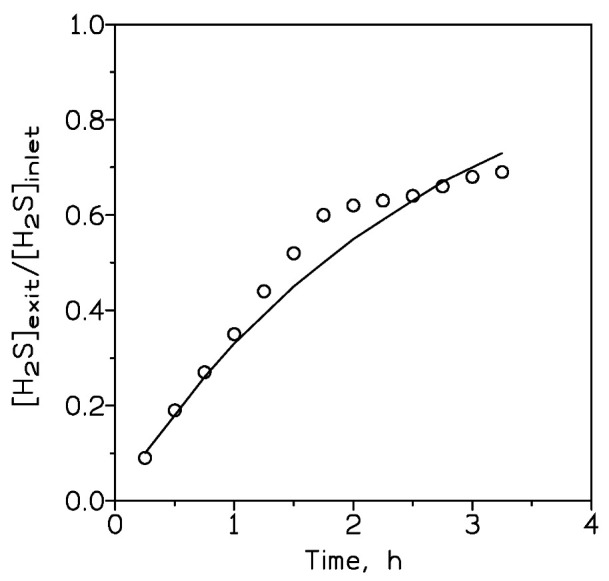
Experimental (circles) and Equation (3) model (line) data of experiments under the following conditions: adsorbent dosage: 4 g. H_2_S dosage: 400 mg. Gas flow: 210 cm^3^/min. Temperature: 20 °C.

The exception to the above fitting is the data resulting from the highest adsorbent dosage (4 g) and lowest flow rate (210 cm^3^/min). In this case, the experimental data best fit an exponential equation in the form:

(3) $$\frac{{\left[S\right]}_{\mathrm{exit}}}{{\left[S\right]}_{\mathrm{inlet}}}=1-{\mathrm{Xe}}^{-\mathrm{Yt}}$$

where X and Y are model parameters and t is equal to zero at the breakthrough point. The fit of the above model to the experimental data is represented in Figure 5, indicating the relative goodness of the model to these non-S-shaped experimental data. The corresponding values of X and Y are 1 and 0.4, respectively. 

Since the model represented in Equation (2) has a tendency to deviate from the experimental data at the extreme points of the S-shape curves, a further refinement in the modeling of the experimental data using the Bohart–Adams model of fixed bed adsorption [23] was conducted.

The model consists of two coupled first-order linear particle differential equations,

(4) $$\frac{\partial a}{\partial t}=-kac,\;\;u\frac{\partial c}{\partial z}=-\rho_{s}kac$$

subject to initial and boundary conditions: *c* = *c*_0_ at *z* = 0, and *a* = *a*_0_ at *t* = 0.

In the above equation, *a* (−) is the adsorption capacity of the bed (the mass of H_2_S that can be adsorbed per unit mass of solid), *c* (the mass unit) is the solute concentration in the gas flow, *k* (the volume/mass and time units) is the adsorption rate constant, $\rho_{s}$ (the mass/volume units) is the bed bulk density (the mass of the solid per unit volume of column units), *u* (the length/time units) is the gas flow velocity, *z* (the length unit) is the axial coordinate along the column, and *t* (the time unit) represented the elapsed time.

The first condition gives the solute concentration in the inlet gas, and the second condition gives the adsorption capacity of the clean bed, i.e., before solute adsorption.

The solution of these equations is:

(5) $$\frac{c\;}{c_{0}}=\frac{\exp\left(kc_{0}t\right)}{\exp\left(kc_{0}t\right)+\exp\left(ka_{0}\rho_{s}z/u\right)-1}$$

(6) $$\frac{a\;}{a_{0}}=\frac{\exp\left(ka_{0}\rho_{s}z/u\right)}{\exp\left(kc_{0}t\right)+\exp\left(ka_{0}\rho_{s}z/u\right)-1}$$

To compare this solution with the experimental breakthrough curve, Equations (5) and (6) must be evaluated by *z* = *H*, where *H* is the bed height. In terms of the gas flow rate, *q* (the mass/time units), and the bed mass, *m_s_* (the mass units). The theoretical breakthrough curve becomes:

(7) $$\;{\left(\frac{c\;}{c_{0}}\right)}_{z=H}=\frac{\exp\left(kc_{0}t\right)}{\exp\left(kc_{0}t\right)+\exp\left(ka_{0}m_{s}/q\right)-1}$$

**Figure 6:**
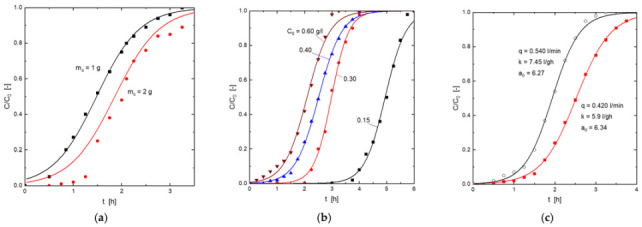
Experimental (symbols) and model (Equations (5) and (6)) (lines) breakthrough curves for the adsorption of H_2_S onto the goethite-based adsorbent. (**a**) Influence of the adsorbent dosage. (**b**) Influence of the H_2_S concentration in the gas mixture. (**c**) Influence of the gas mixture flow.

*K* and *a_0_* values were determined (Table 5) by the least square fitting of the experimental data to Equation (7). The fit for the various operating conditions is shown in Figure 6. It can be seen that the resolution of this model adequately represented the uptake of H_2_S onto this goethite-based adsorbent. 

Furthermore, the bed adsorption efficiency can be calculated using:

(i) in an instantaneous time as:

(8) $$E_{ins}\left(t\right)=1-\frac{c_{t}}{c_{0}}$$

(ii) or in an average form over a time t as:

(9) $$E\left(t\right)=\frac{1}{t}\int_{0}^{t}\left(1-\frac{c}{c_{0}}\right)dt$$

Inserting Equation (5) in Equation (10) resulted in:

(10) $$E\left(t\right)=1-\frac{1}{kc_{0}t}\ln\frac{\exp\left(kc_{0}t\right)+\exp\left(ka_{0}m_{s}/q\right)-1}{\exp\left(ka_{0}m_{s}/q\right)}$$

**Figure 7:**
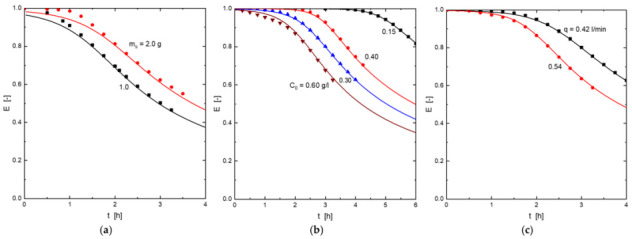
Experimental (symbols) and model (Equation (10)) (lines) column efficiencies. (**a**) Influence of the adsorbent dosage. (**b**) Influence of the H_2_S concentration in the gas mixture. (**c**) Influence of the gas mixture flow.

Figure 7 represented the different experimental (symbols) and calculated (from Equation (10)) efficiency values for the different operational conditions used in the present work. An excellent fit between the values was found.

## 3. Materials and Methods

### 3.1. Adsorbent

**Figure 8:**
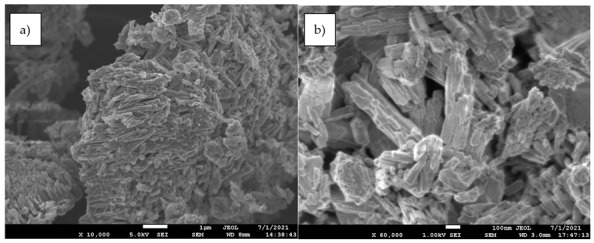
Goethite crystals observed by scanning electron microscopy (SEM). (**a**) general appearance of grain and (**b**) magnification (×60,000).

The adsorbent was supplied by a Spanish mine and consisted of micronized goethite particles (Figure 4a and Figure 8), which were obtained by extraction and micronization. The particle size distribution of the goethite particles was determined as D_50_ = 7.81 µm and D_90_ = 32.7 µm. It was used without further modifications.

### 3.2. Adsorption Tests

**Figure 9:**
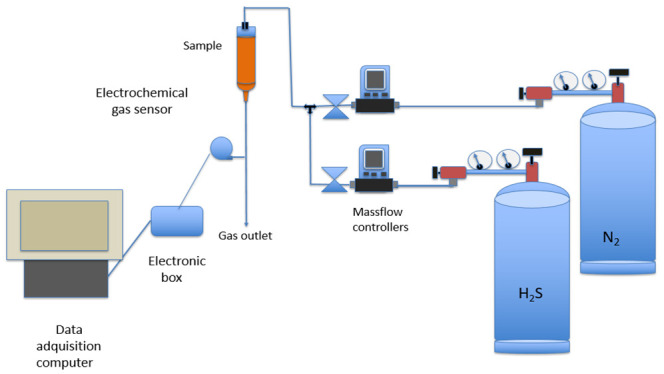
Schematic view of the setup used to investigate H_2_S uptake onto the adsorbent.

The adsorption of H_2_S onto the material was investigated at room temperature (20 °C) using the experimental setup shown in Figure 9 and described below. It consisted of a column containing the weighed adsorbent samples from which the gas samples were flown upside down. The various H_2_S concentrations in the gas mixture (H_2_S and N_2_ from separated bottles) were controlled by a Bronk Horst HI-TEC F-201C-FA-22-V (Bronkhorst High-Tech B.V., Nijverheidsstraat, The Netherlands) mass controller in the case of H_2_S, or an Alicat Scientific MC-500SCCM-D (Alicat Scientific, Tucson, AZ, USA) mass controller for N_2_. In the case of the H_2_S controller, the flow range varied between 0 and 200 mL/min, whereas in the case of the N_2_ controller, the flow varied between 0 and 500 mL/min.

Exiting H_2_S concentration from the column was measured online by an Amphenol SGX Sensortech 4 series electrochemical gas sensor (Sensortech, Corcelles-Cormondreche, Switzerland). Data were stored using a data acquisition computer running a specific software (SGX ECVQ-EK3 Gas Sensor Evaluation KIT V 2.1.0, Sensortech, Corcelles-Cormondreche, Switzerland).

### 3.3. Characterization Techniques

A Field Emission Scanning Electron Microscope equipped with an Energy Dispersive Spectroscopy analyzer (FE-SEM-EDS, S-4700type I-Hitachi, Tokyo, Japan) was used to investigate the morphology and microstructure of the adsorbent. This adsorbent, in powder form, was placed manually on a graphite foil and coated by Au sputtering for low FE-SEM magnification observations, whereas EDS and Pt sputtering were used for high magnification observations.

The particle size distribution was determined by laser scattering using a Master Sizer 2000 (Malvern, UK) equipment.

X-ray diffraction (XRD, D8 Advance, Bruker AXS GmbH, Bremen, Germany) analyses were performed with a copper anode (CuKα1 λ = 0.15418 nm) working at 40 kV and 40 mA. Determinations were conducted on samples rotating at 15 rpm in the interval 10–70° (2θ) and X-ray patterns were acquired with a step/size of 0.02° and time/step of 2 s.

The chemical analysis was carried out by means of wavelength dispersion using an X-ray Fluorescence (XRF) technique with PANalytical equipment (MagicX PW-2424, Axios, PANalytical, Malvern, UK) with an Rh anode RX tube (SUPER SHARP, Malvern Panalytical, Malvern, UK) and generator of 2.4 KW.

## 4. Conclusions

As a resource of renewable energy, the cleaning of biogas using a cheap and readily available Spanish raw material, such as a goethite-based adsorbent, and under dynamic conditions is demonstrated. The adsorption of toxic H_2_S onto the adsorbent is dependent on various experimental variables such as adsorbent dosage, H_2_S concentration in the gas mixture, and gas mixture flow. The breakthrough point (the time at which the [H_2_S]_exit_/[H_2_S]_inlet_ relationship is equal to 0.05) increases with the increase of the adsorbent dosage from 1 g to 4 g, the decrease of the initial H_2_S concentration in the gas mixture from 600 mg to 150 mg, and the decrease of the gas flow from 540 cm^3^/min to 210 cm^3^/min.

Several theoretical models are adopted to represent the column performance. The models, with the corresponding model parameters properly identified, predict reasonably well the experimental breakthrough curves. Moreover, a model equation is derived to show the column efficiencies under the various experimental conditions used in this investigation.

## Figures and Tables

**Table 1:** H_2_S loading at the breakthrough point using various adsorbent dosages.

Adsorbent Dosage, g	Breakthrough Point, h	[H_2_S], g/g
1	0.50	2.5
2	1.00	2.5
4	2.10	2.6

**Table 2:** H_2_S loading at the breakthrough point at various inlet H_2_S dosages.

[H_2_S], mg	Breakthrough Point, h	[H_2_S], g/g
150	3.90	3.6
300	2.25	4.2
400	1.50	3.8
600	0.50	2.3

**Table 3:** H_2_S loading at the breakthrough point at various gas flows.

Gas Flow, cm^3^/min	Breakthrough Point, h	[H_2_S], g/g
210	2.10	2.6
420	1.50	3.8
540	0.75	2.4

**Table 4:** Parameters of Equation (2).

Adsorbent Dosage, g	Gas Flow, cm^3^/min	H_2_S Dosage, mg	K, h^−1^	t_50_, h	r^2^
1	210	400	2.2	1.47	0.9977
2	210	400	1.8	1.83	0.9889
4	420	600	2.5	2.13	0.9942
4	420	400	2.4	2.52	0.9918
4	420	300	2.7	2.96	0.9919
4	420	150	2.7	4.96	0.9927
4	540	400	3.1	1.94	0.9990

**Table 5:** Parameters of the Equations (5) and (6).

Experimental Condition	k, L/g·h	a_o_, g/g	Comment
1 g adsorbent 2 g adsorbent	5.6 5.6	7.5 4.7	(1)
150 mg H_2_S dosage 300 mg H_2_S dosage 400 mg H_2_S dosage 600 mg H_2_S dosage	17.6 10.5 5.9 4.0	4.7 5.6 6.3 7.9	(2)
420 cm^3^/min gas flow 540 cm^3^/min gas flow	5.9 7.5	6.3 6.3	(3)

(1) Data corresponding to Figure 6a. (2) Data corresponding to Figure 6b. (3) Data corresponding to Figure 6c.

## Data Availability

Not applicable.
